# Differences in Decision-Making Behavior Between Elite and Amateur Team-Handball Players in a Near-Game Test Situation

**DOI:** 10.3389/fpsyg.2022.854208

**Published:** 2022-04-05

**Authors:** Matthias Hinz, Nico Lehmann, Norman Aye, Kevin Melcher, J. Walter Tolentino-Castro, Herbert Wagner, Marco Taubert

**Affiliations:** ^1^Department of Sport Science, Faculty of Human Sciences, Institute III, Otto von Guericke University Magdeburg, Magdeburg, Germany; ^2^Department of Neurology, Max Planck Institute for Human Cognitive and Brain Sciences, Leipzig, Germany; ^3^Department of Performance Psychology, Institute of Psychology, German Sport University Cologne, Cologne, Germany; ^4^Department of Sport and Exercise Science, Paris Lodron University Salzburg, Salzburg, Austria; ^5^Center for Behavioral and Brain Sciences (CBBS), Otto von Guericke University Magdeburg, Magdeburg, Germany

**Keywords:** sensorimotor decisions, expertise, decision time, motor responses, perceptual-cognitive skills

## Abstract

Athletic features distinguishing experts from non-experts in team sports are relevant for performance analyses, talent identification and successful training. In this respect, perceptual-cognitive factors like decision making have been proposed to be important predictor of talent but, however, assessing decision making in team sports remains a challenging endeavor. In particular, it is now known that decisions expressed by verbal reports or micro-movements in the laboratory differ from those actually made in on-field situations in play. To address this point, our study compared elite and amateur players’ decision-making behavior in a near-game test environment including sport-specific sensorimotor responses. Team-handball players (*N* = 44) were asked to respond as quickly as possible to representative, temporally occluded attack sequences in a team-handball specific defense environment on a contact plate system. Specifically, participants had to choose and perform the most appropriate out of four prespecified, defense response actions. The frequency of responses and decision time were used as dependent variables representing decision-making behavior. We found that elite players responded significantly more often with offensive responses (*p* < 0.05, odds ratios: 2.76–3.00) in left-handed attack sequences. Decision time decreased with increasing visual information, but no expertise effect was found. We suppose that expertise-related knowledge and processing of kinematic information led to distinct decision-making behavior between elite and amateur players, evoked in a domain-specific and near-game test setting. Results also indicate that the quality of a decision might be of higher relevance than the required time to decide. Findings illustrate application opportunities in the context of performance analyses and talent identification processes.

## Introduction

Features that set apart the sports experts from the non-experts have been a topic of considerable research efforts during the last years. In this regard, it has been proposed that physical (e.g., anthropometric), physiological, psychological, sociological, technical, and tactical factors discriminate between athletes of different levels of expertise (Burgess and Naughton, 2010; Sarmento et al., 2018; Piggott et al., 2020). Most emphasis in research has been placed on studying physical aspects and biomotor abilities (such as speed, strength, power, agility, and endurance), although recent studies suggest that the predictive validity of these factors regarding performance and talent identification is limited, especially when they are looked at in isolation (Wagner et al., 2016, Wagner et al., 2019, 2020; Bennett et al., 2018; Bergkamp et al., 2019). This might be due to the fact that these particular predictors are not representative enough with respect to the contextual constraints of on-field behavior (Bergkamp et al., 2019). To overcome this problem, multidisciplinary approaches have been suggested to diagnose future performance and talent. Particularly, the need to focus more on the psychological and perceptual-cognitive components of athletes has been highlighted in recent studies (Bangsbo, 2015; Woods et al., 2016; Bennett et al., 2018; Bergkamp et al., 2019; Sherwood et al., 2019; Piggott et al., 2020). Indeed, mounting evidence suggests that perceptual-cognitive skills such as decision making constitute a performance-discriminating component in team-sports (Mann et al., 2007; Travassos et al., 2013; Silva et al., 2020; Ashford et al., 2021).

It is against this background that the development and evaluation of tests to diagnose perceptual-cognitive skills and expertise in athletes has gained more and more attention in the last years. A commonly used approach to study sport-specific decision making is the so called temporal occlusion paradigm (Jones and Miles, 1978). Essentially, studying decision making with this approach involves presenting video clips of selected game sequences on a screen, and subjects watch these clips while usually being in a sitting or standing position (Travassos et al., 2013 for details). After the end of the video sequences, subjects are mostly required to verbalize their intended response for the game situation in question, or to verbalize their generated options (Johnson and Raab, 2003; Raab and Johnson, 2007; Raab and Laborde, 2011). Decision making in real life evolves from a complex and uncertain context (especially in team-sports), requiring athletes to constantly process information while acting under time and information constraints (Travassos et al., 2012; Kinrade et al., 2015). Against this background, it appears problematic to not consider the specific environment in which the players actually perform, and particularly to neglect the impact of sensorimotor interactions in decision making (Burk et al., 2014; Raab, 2014). Recent examinations in netball (Bruce et al., 2012), and soccer (van Maarseveen et al., 2018a) clearly suggest that the decision-making performance in perceptual-cognitive tests (using verbal reports, button press, or micro-movements) differs from actual real-world decision-making performances, thus hampering the ecological validity of findings (Araújo et al., 2007; Ashford et al., 2021). Taken together, these studies highlight that assessing verbal or micro-movement responses (Travassos et al., 2013) might be not sufficient to predict on-field performance, let alone to detect talents. Notwithstanding, there is also evidence in perceptual-cognitive research which showed that uncoupled perception-action responses, given either verbally or by keystroke, are similar (Farrow et al., 2005) or even more accurate (Ranganathan and Carlton, 2007) than coupled perception-action responses, requiring sport-specific action responses. It was also found that when observers are static in computer-based experiments, the motor regions of the brain are still linked to the perceptual information picked up (Aglioti et al., 2008). In team-handball (Huesmann et al., 2021), compared the anticipation performances of advanced, intermediate, and novice team-handball goalkeepers in a perception-action artificial (verbal responses) and simulated (motor responses) condition with. The authors revealed overall superior performances (higher prediction accuracies) in the artificial, verbal response condition, outlining that the evidence regarding the necessity of the involvement of motor components seems mixed. However, when capturing expert performance in decision making, expertise effects are most pronounced when the participants actually performed actions under *in situ* task constraints (Travassos et al., 2013) in realistic test paradigms under field conditions (Mann et al., 2007).

Since the discrepancy between decision making in decoupled vs. coupled processes of perception and action in task designs has only recently become known, there are only a few studies assessing decision making in near-game test conditions with requirements to perform a sport action (Travassos et al., 2013). One notable example is the study by Magnaguagno and Hossner (2020) in team-handball, which investigated decision making in a performer environment by using virtual reality. Specifically (Magnaguagno and Hossner, 2020), presented 1 vs. 1 video sequences between a defending teammate and a left or right back attacker, respectively, ending in either a successful or a lost defense action of the teammate. The participants were put into an assisting role as a defender next to the 1 vs. 1 situation. Depending on the teammates’ either weak or strong defending behavior, participants had to decide (based on their anticipatory performance) whether to move sideways for tackling the attacker (in case the weak teammate has lost his duel), or whether to stay in the passive position (in case the strong teammate successfully defended the attacker). The authors found expertise effects in response correctness, showing that expert players responded more appropriately on a lost 1 vs. 1 duel of the respective defending teammate than the near-expert players. However, response correctness simply based on the decision whether to stay passive or to tackle. Further options for defense actions, e.g., provoked by additional varying attack sequences, were not regarded. Also, the respective time of responses (decision time) was not recorded, even though decision time is thought to be an important metric of decision making (Vaeyens et al., 2007; Raab and Laborde, 2011; Ratcliff et al., 2016; Seale-Carlisle et al., 2019).

In the present study, we investigated whether decision-making in a near-game performer environment would differ between expert and near-expert athletes. To this end, we used a team-handball specific sensorimotor decision-making task with varying attack sequences based on the temporal occlusion approach (see Jones and Miles, 1978 for details). We have previously shown that this test setup is sufficiently reliable to study decision making (Hinz et al., 2021), and by comparing experts vs. near-experts we now undertake the next step to discover the potential usefulness of this test to distinguish expert vs. non-expert performance. This test setup involves both domain-specific motor responses (as compared to, for example, button press) and the opportunity to choose among various response options (as compared to “either-or decision making”). We also recorded decision times which allowed us to study whether expert and near-expert players would initiate a different defense action (e.g., a “proactive” behavior like tackling vs. a “passive” behavior like blocking) in response to identical visual information, and whether there are differences in the accompanying decision times. In order to use representative task constraints (Travassos et al., 2013), we also investigated the decision-making performances in right- as well as left-hander attack sequences, due to handedness advantages in favor of left-handers in sport (Hagemann, 2009; Loffing et al., 2015).

## Materials and Methods

Carrying out between-group comparisons with multiple choices for responses entails difficulties in estimating *a priori* effect sizes. Therefore, our sample size recruitment complied with sub-sample sizes from earlier between group investigations in this field (e.g., Raab and Johnson, 2007; Zoudji et al., 2010; Bruce et al., 2012).

The sample of participants consisted of 44 male team-handball players (*M*_*age*_ = 19.11 years; SD = 6.56 years). Two teams (*n* = 22; *M*_*age*_ = 17.59 years, SD = 3.67 years) were recruited of a professional youth academy of a first league team-handball club of the German Handball Federation. All players competed in the highest possible league within their age category. Players of these teams performed a minimum of 14 h training per week with one competition match at weekends. All athletes practiced team-handball for at least 8 years. Based on the recommendations of Swann et al. (2015) how to classify expertise level in sports science, players of the two teams can be considered as elite level players. The players of the other two teams (*n* = 22; *M*_*age*_ = 20.71, SD = 8.54) were recruited from non-professional, local league teams within their age categories. All athletes performed 4 h of training per week with one competition match at weekends, and players practiced team-handball between 2 and 22 years. According to the definition of Room (2010), who defines a player, “who takes part in sport for pleasure, as distinct from a paid professional” as amateur player, the athletes of these two teams can be considered as amateur level players. Differences in age between both groups were not significant (*p* = 0.952).

The experiment was conducted during the first half of the championship season 2020/21, in October and November. At that time, all teams had a normal weekly training and match schedule, without being affected or restricted by any local or federal COVID-19 regulations. During the test, participants were instructed to perform with a maximum effort. Injuries lead to exclusion of the study. Prior to their participation, all participants and legal guardians were informed about the purpose, risks, and benefits of the study. All participants had to give a written informed consent before the first test day. Participants were not identifiable from the test results. The study protocol was approved by the local ethics committee from the Otto von Guericke University Magdeburg and met the requirements of the Declaration of Helsinki and its later amendments.

All tests were conducted on the contact plate system SpeedCourt^®^ (Q12 PRO mobile, GlobalSpeed, Hemsbach, Germany) which enables sport-specific motor responses to temporally occluded videos. As a basis for profound interpretations of envisaged results, we used the test setup from a previous study (Hinz et al., 2021), which was introduced and checked for basic psychometric properties (reliability), using four team-handball specific attack actions for intra- (cross-sectional) and inter-session (longitudinal) agreement of the motor response choices and times. Significant Cohen’s (0.44–0.54) and Fleiss’ (0.33–0.51) kappa statistics (Fleiss and Cohen, 1973) revealed moderate agreement level of motor responses. Please refer to this paper for detailed explanations regarding test construction and item analyses. In the study at hand, we used the identical test setup and video stimuli, along with the same mapping of the four choice responses (forward/tackling response; sideways left or right movements; blocking/passive behavior) to the contact plates on the SpeedCourt^®^.

The experimental scenario consists of *Breakthrough*, *Standing throw*, *Jump throw*, and *Pass* videos, which were temporally occluded within a general time frame of *ball was passed to attacker* (*t*_6_) and *obvious end of attack* (*t*_0_), with time intervals of 200 ms (*t*_6_ = −1200 ms, *t*_5_ = −1000 ms, *t*_4_ = −800 ms, *t*_3_ = −600 ms, *t*_2_ = −400 ms, *t*_1_ = −200 ms, *t*_0_ = 0 ms). The duration of each video clip was not longer than 2 s (stopping at *t*_0_). Dummy trial videos, showing too ambiguous attack actions for an appropriate defense response, were included in the test scenario, aiming at avoiding expectation effects in response behavior (Anderson, 1983). Due to handedness advantages in favor of left-handers in sport (Hagemann, 2009; Loffing et al., 2015), all video clips were mirrored.

The videos were sized 1280 × 720 pixels (width × height) and the test scenario was implemented by using *Lazarus* (Version 2.0.10) software. In total, 112 right- and left-handed attack video clips were presented to the subjects during the measurement procedures.

The test procedure always started on the marked 7 m line on the central contact plate of the SpeedCourt^®^. In this starting position, a 3 s countdown appeared on the screen, followed by a video stimulus showing an attack action. The aim was to respond as intuitive and as realistic as possible after the video presentation ended. Subjects were then returned to the starting position to prepare for the next countdown. Subjects were instructed that the motor response to a video stimulus should replicate their first intuition for a defense response that came to their mind while watching the video. Too early or unintended given responses were marked for later exclusion. No information about decision performance or the remaining number of videos was provided to the subjects.

In relation to the Bayesian integration framework (Vilares and Kording, 2011; Gredin et al., 2020), all subjects received the same team-handball specific instructions (stable priors) about the attacker’s action tendencies, the defense tactics, and the match status. Stable context priors *via* action tendencies were provided, meaning that the center back player in the video can be considered as an allrounder or playmaker, being able to put high pressure on the defense through a variety of long and near range standing and jump throws, strong one-on-one actions, and high-quality passing. Tactical priors were also supplied to the subjects. More specifically, they were instructed to put themselves in the position of the central block defender in a classic man-to-man defense without teammates, or other opponents than the attacker in the video/situation. The match status was pre-specified as the 50th minute of play (of 60 min in total) and the game score was tied.

Following the instructions, subjects performed 10 familiarization trials, showing a selection of occluded attack actions in randomized order. After familiarization, the test started with a block of right-hander video stimuli followed by a left-hander block, interspersed by a short break of approximately 5 min. Within each block, the videos were presented in quasi-randomized order, starting at occlusion *t*_6_ (fewest information) and ending at *t*_0_ (full information) videos. The test duration was approximately 35 min.

### Analysis

All data used in this study was recorded from the contact plates of the SpeedCourt^®^ system. Dependent variables were response frequency (categorical) and decision time (in ms). A motor response was registered when leaving a contact plate and entering a new/the same contact plate. Decision time was defined as the time elapsed from the end of the video presentation to the beginning of the motor response (force on plate > 80 N).

We applied an outlier detection procedure based on decision time data, as proposed by Leys et al. (2013). Specifically, we started by calculating the absolute deviation around the time sample median for each occlusion point in each action. A moderately conservative rejection criterion of 2.5 times the median absolute deviation (MAD) below or above the median was defined. In other words, individual time data was categorized as outliers if their time value fell outside the predefined rejection criterion. If outliers of data points were detected, all related variables (i.e., choice of motor response, decision time) of the respective case were discarded from further statistical analysis.

Unless otherwise stated, the Statistical Package for the Social Sciences Version 26 (SPSS Inc., Chicago, IL, United States) was used for inferential statistics in the following analyses. Statistical tests of significance carried out throughout the manuscript were performed two-sided, and the significance level was set to *p* < 0.05.

For the characterization of distinctions in decision making behavior between elite and amateur players, we compared the frequencies of the occurrence of each motor response (i.e., forward/tackling; passive/blocking; sideways right; sideways left) at each occlusion point by means of a Chi-squared test. The magnitude of Chi-square-based associations was evaluated using the effect size Phi (φ) (Kim, 2017). Phi was calculated by dividing the Chi-square value by the sample size *n* and then taking the square root, yielding a value ranging from −1 to 1. The magnitude of φ can be interpreted using the following thresholds (Cohen, 1988; Kim, 2017): 0.1 < | φ| < 0.3 “small,” 0.3 < | φ| < 0.5 “medium,” and | φ| > 0.5 “large” effect. Note that negative values for φ denote higher frequencies in the elite player group and reverse for positive values.

To summarize evidence over the seven Chi-squared tests (i.e., occlusion points *t*_6_–*t*_0_) belonging to each motor response and each action, partial two-sided *p*-values were combined into a single global *p*-value using Fisher’s Chi-square combination (Fisher, 1932):


$$\chi^{2}=-2\,\sum_{i=1}^{k}\ln\left(p_{i}\right)\,$$

In case the combined null hypothesis of no between-group difference whatsoever is rejected, one can conclude that at least one of the partial null hypotheses is false. Put another way, Fisher’s combination allows to combine multiple pieces of evidence to yield a style of meta-analytic result. Odds ratios (OR) with a 95% confidence interval (CI) were calculated (as described by Bland and Altman, 2000) by pooling all responses in each occlusion in the single attacks, in order to obtain summarized effect sizes of Chi-square combinations. Note that Fisher’s combination was only applied if the direction of between-group differences was consistent across all occlusion points. The above was done using the *poolr* package (Cinar and Viechtbauer, 2021) running in *R* v3.6.1 (R Core Team, 2013).

In our previous study (Hinz et al., 2021), as expected, we observed quicker response times as a function of increasing amount of visual information. However, it remains to be determined whether experts and amateurs differ with respect to decision times. To this end, we subjected decision time data to a 2 (elite and amateur level) by 7 (occlusion time point *t*_6_–*t*_0_) repeated measures analysis of variance (ANOVA). Before ANOVAs were calculated, all data was checked for normality using the Kolmogorov–Smirnov test.

## Results

**FIGURE 1:**
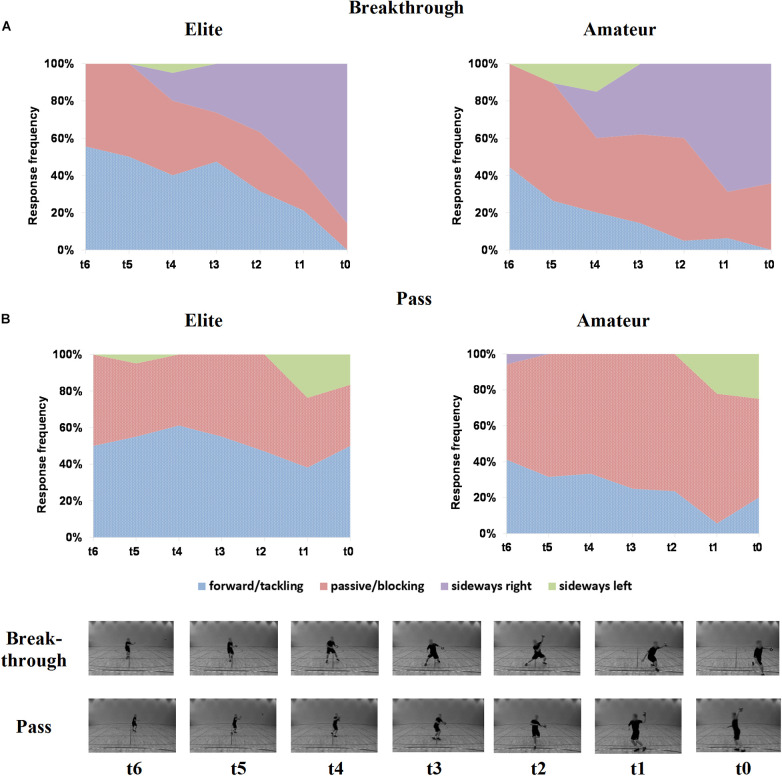
Illustration of significant different frequency distributions of motor responses in left-handed Breakthrough **(A)**, and left-handed Pass **(B)** of elite (left) and amateur players (right). Stacked area graphs show occlusion points (*x*-axis) and (relative) response frequency (*y*-axis). Dotted areas denote significant frequency distribution differences between groups. Screenshots of each video (and its constituent occlusions) are shown at the bottom.

Significant between-group differences of the response frequency distributions are shown in Figure 1. Significant effects of expertise were present in the left-handed Breakthrough and Pass only. Full illustrations of response frequency and distribution in all attacks, as well as individual Chi-square statistics, are provided in Supplementary Figures 1, 2 and Supplementary Tables 1, 2.

Irrespective of group, visual inspection of frequency distributions shows dynamically changing response patterns over occlusion points, most likely due to the varying amount of visual information provided about the attacker’s action. As indicated by significant results of Fisher’s combination, the elite players responded significantly more often with forward/tackling movements in Breakthrough [χ^2^(14) = 25.06, *p* = 0.033, OR = 2.76, CI = 1.54–4.95] and Pass [χ^2^(14) = 37.19, *p* = 0.001, OR = 3.00, CI = 1.78–5.04]. On the contrary, amateur players instead showed a more frequent use of passive/blocking in Pass [χ^2^(14) = 28.28, *p* = 0.013, OR = 2.70, CI = 1.64–4.46] (see Supplementary Table 2). Of note, especially regarding single occlusion points with comparably few visual information, elite players use more frequently forward/tackling in Breakthrough [*t*_3_: χ^2^(1) = 5.20, *p* = 0.023, φ = −0.36]. In Jump throw, elite players use more frequently forward/tackling in *t*_4_ [χ2(1) = 4.64, *p* = 0.031, φ = −0.36], but switching to more frequent passive/blocking responses at *t*_3_ [χ2(1) = 4.50, *p* = 0.034, φ = 0.35]. Another significant between-group difference was observed in Jump throw, where amateur players responded more often with a sideways right move [*t*_4_: χ2(1) = 3.90, *p* = 0.048, φ = 0.33] (see Supplementary Table 2).

**FIGURE 2:**
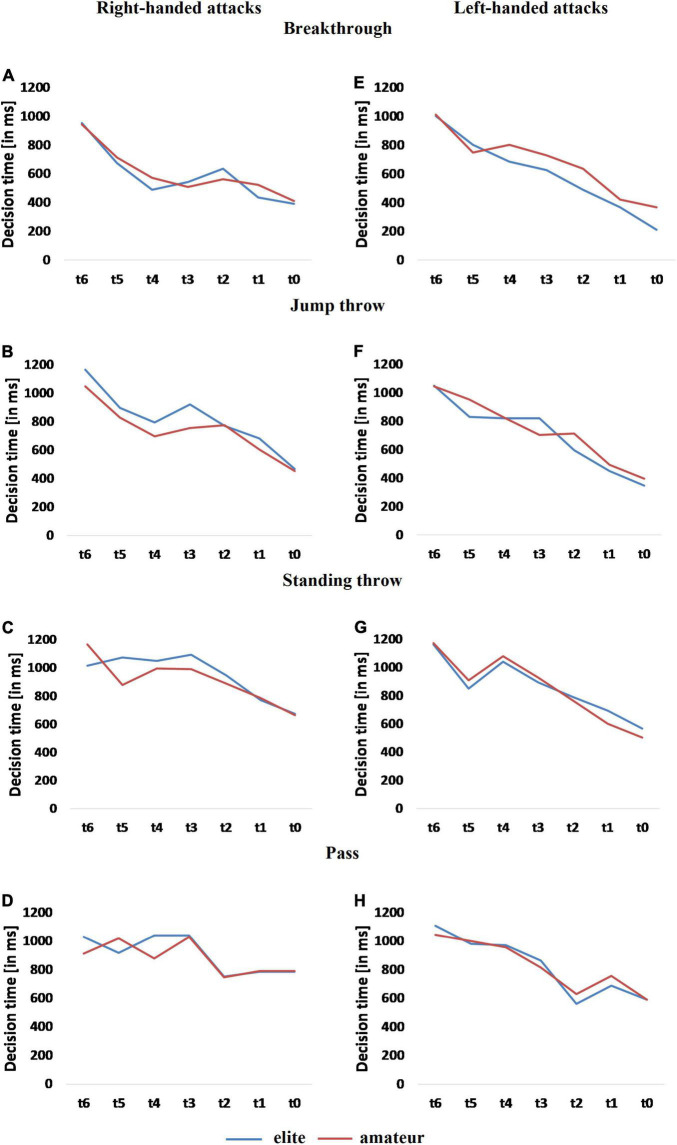
Comparisons of aggregated decision times for all responses between elite and amateur players in right- **(A–D)** and left-handed **(E–H)** attacks.

As expected, based on our previous study (Hinz et al., 2021), faster decision times occurred with increasing visual and kinematic information of the attacker in both groups (Figure 2). This is evidenced by significant results for the main effect “occlusion” in the repeated measures ANOVA in right-handed [Breakthrough: *F*(6, 90) = 4.42, *p* < 0.001; Jump throw: *F*(6, 78) = 10.34, *p* < 0.001; Standing throw: *F*(6, 96) = 9.52, *p* < 0.001; Pass: *F*(6, 96) = 6.51, *p* < 0.001] and left-handed [Breakthrough: *F*(6, 84) = 27.48, *p* < 0.001; Jump throw: *F*(6, 96) = 32.51, *p* < 0.001; Standing throw: *F*(6, 120) = 18.67, *p* < 0.001; Pass: *F*(6, 114) = 17.29, *p* < 0.001] actions. Between-group comparisons of the main effect “expertise,” however, revealed no significant effects. A significant group-by-occlusion [*F*(6, 96) = 2.33, *p* < 0.038] interaction was detected in the right-handed Standing throw. A detailed overview of ANOVA statistics is presented in Supplementary Table 3.

## Discussion

Considering the need of motor responses in expert decision-making research (Travassos et al., 2013), the current study compared the decision-making behavior between elite and amateur team-handball players, by using sport-specific motor responses in representative near-game test situations. To do so, we measured the frequencies of selected responses, which were given as a team-handball specific defense action on occluded video sequences showing varying attack actions. Additionally, we were interested in the decision time each player required to initialize the respective response selected.

Regarding response frequency, we identified significant effects of expertise in the left-handed Breakthrough and Pass, where elite players demonstrated an overall significant preference to respond with forward/tackling movements on both attack actions. The amateur players, however, preferred to stay rather passive or blocking in Pass. More specifically, preferences for forward/tackling response or passive/blocking responses by the elite players in single occluded time points were also found (at *t*_4_ in left-handed Breakthrough and left-handed Jump throw, and *t*_3_ in left-handed Jump throw). Interestingly, the amateur players demonstrated significant more frequent sideways right responses in the left-handed Jump throw attack. Taken together, the differing frequencies of selected responses from both player groups suggest an expertise-dependent decision-making behavior. Expert effects in our study align with previous motor experiments in decision-making research in team-handball (Raab, 2003; Magnaguagno and Hossner, 2020), and extend them by new insights into how elite and amateur players differ in their tactical understanding of defending when limited visual information is provided.

Regarding decision time, we detected a reciprocal decrease of the time for a decision with increasing kinematic information in the presented attack actions (as found in Hinz et al., 2021). Despite the overall significance of this effect in all of the attacks, an expertise effect in decision time did not appear at all. To the best of our best knowledge, comparable decision (or response) time data from related motor experiments in team-handball using near-game environments do not exist (Bonnet et al., 2020). Previous non-motor experiments (Raab and Laborde, 2011) using offense sequences found expert players to decide better and faster, however, a similar study investigating the influence on mood on decision making found no expertise effect in decision time (Laborde and Raab, 2013). Likewise, the decision time data in our study was also not able to discriminate between groups. The comparability to both of the mentioned studies is to be seen with care, due to the specificity of the offense or defense situations the players were tested in, and due to the methodological aspect of sensorimotor responses in our test instead of verbal reports as responses.

### Complex Sensorimotor Decisions Can Distinguish Expertise Levels

In order to classify our findings regarding decision-making behavior, to the best of our knowledge, the virtual reality study of Magnaguagno and Hossner (2020) is one of just a few comparable studies using a sport-specific motor approach to assess defense behavior in team-handball (Magnaguagno and Hossner, 2020; Hinz et al., 2021). Against our approach with four different attack actions in combination with multiple-choice responses, the patterns of play in their video sequences remained stable (lost or won 1 vs. 1 duel of teammate), and response choices were not prespecified. Similar to our expertise effects in decision making, the authors detected significant expert advantages in the correctness of the given motor responses, but response times of the tactical decisions were not regarded, and differentiations of response outcomes regarding handedness of the attacker were also not undertaken. However, the comparability of results from both studies is partially restricted by the assisting role for the participants in the 2 vs. 2 group tactic situation in the Magnaguagno and Hossner study, in contrast to our active 1 vs. 1 situation. Nevertheless, the distinct anticipatory decision-making abilities between experts and near-experts in the mentioned study coincide with our findings of stronger preferences for more offensive-orientated defense actions such as forward movement/tackling of the elite players. The expert players in the Magnaguagno and Hossner study equally demonstrated a significant more frequent tackling behavior.

An interpretation for the evident preferences of elite players for enhanced offensive movements (forward/tackling) in the left-handed attack actions (Figure 1) could be that higher performing athletes use kinematic cues in anticipatory processes differently compared to their lower performing counterparts, as stated in research so far (Jackson et al., 2006; Johnston and Morrison, 2016). Put another way, based on the perception of kinematic cues throughout the attackers’ movement, elite players could judge this visual information differently, resulting in an altered conversion into a motor response. Another explanatory approach could be found in the impact of the provided context priors to the players (Gredin et al., 2020) in combination with perceived kinematic cues of the attackers’ actions. That elite players make different decisions than amateurs might be due to their expert knowledge and experience with the specified defensive tactics, the match status and/or the minute of play. The test instructions provided explanations about a man-to-man defense system players, and certain rules within this defense system apply, taught in basic practice lessons from early team-handball ages on Pabst and Scherbaum (2018). With increasing age and expertise level, elite players practice more often and compete higher, therefore learning and adapting defense systems on a higher competition level. We therefore assume that the elite players not only perceive the kinematic information of the attacking players’ actions but also taking the increased risk for a wrong decision into account, which is enhanced by the tied game score and the approaching end of the match, subsequently making different tactical decisions than amateur players.

Our study also demonstrated assorted decision-making behavior in right- and left-handed attacks, which transfers the hand-specificity effects from recent embodied choice experiments (van Maarseveen et al., 2018b) into our setting. Previous research on hand-effects in team-handball found evidence for the lack of familiarity with less frequent left-handed opponents (Baker et al., 2013; Loffing et al., 2015), and the dependence on an observers’ domain-specific skill to identify the opponent’s unfolding action (Loffing et al., 2015). The results obtained from the present study suggest that laterality effects on handball-specific decision making can also be observed in test settings involving motor responses.

### Elite Players Invest More Time for Different Decisions

Expectedly, the decision times generally decreased with an increasing amount of visual information as detected in previous team-handball experiments (Raab and Johnson, 2007; Raab and Laborde, 2011; Laborde and Raab, 2013; Loffing et al., 2016; Cocić et al., 2021). Unexpectedly, the non-existent differences in decision time between elite and amateur players are in contrast to previous team-handball studies that determined experts to make better and faster decisions (Raab and Laborde, 2011). It may be assumed that this discrepancy is at least in part related to differences in the experimental setups between studies (e.g., response methods, sample sizes, sub-sample expertise). Providing responses verbally (or *via* keystroke) might yield different outcomes as compared to motor responses in a performer environment setting, as presented in the literature (Ranganathan and Carlton, 2007; Huesmann et al., 2021). At this point, we can only speculate about the reason for this apparent discrepancy.

Considering motor response times in multi-choice tasks (Ratcliff et al., 2016) suggests an increase in insights in cognitive-motor differences between domain-specific expert levels. In their meta-analysis (Travassos et al., 2013), found a moderating effect of requisite responses on decision time in decision-making experiments (*p* < 0.001). A closer inspection on expertise difference for decision time under *in situ* conditions in their analyses revealed non-significant differences between performance level (*p* = 0.82), provided solely by two appropriate studies in the review. Despite the small number of studies, these findings are in agreement with the non-discriminating effect of decision time in our results.

With reference to our complex decision-making setup, and the expertise-related decision-making behavior, we assume that *dynamic inconsistency* mechanisms affect the characteristics of individual decisions in our sample. *Dynamic inconsistency* (Raab, 2003; Raab and Johnson, 2007) explains the tendency to deliberately select a better response option after a first intuitive option that came to an athletes’ mind. In this regard, equal decision times might be a consequence of expertise-related top–down (cognitive control of sensory processing) and bottom–up (absence of cognitive control in sensory processing) processes (Raab, 2014) in experts compared to amateurs. We conjecture this deliberate (also considered as corrective) decision-making behavior to be the decisive one that may impact the required time to choose the final decision, and subsequently the decision outcome itself. It can thus be conceivably hypothesized that the perceived kinematic information of the attackers’ unfolding action lead to a first intuitive decision preference in both the elite and amateur players (Raab and Laborde, 2011), preparing a motor response tendency toward the attacker (Raab, 2014). But with further kinematic cues in the ongoing time-motion course (occlusions) of the attackers’ action, the additional perceived information seem to be judged differently by the elite players compared to the amateur players. The elite players may use recent kinematic information (bottom-up process) for a short-term switch to a more appropriate response, evoked by additional time investments. Such a tendency to switch may depend on accumulated, competitive experiences that equip experts with domain-specific knowledge about situation-specific optimal choices (Raab, 2014, 2020). The suspected corrective and deliberate decisions in elite players in our study differ to some extent from the faster, intuitive decisions of experts in the reported literature (Raab and Johnson, 2007; Raab and Laborde, 2011; Laborde and Raab, 2013). Nevertheless, the comparison of the findings within this context has to be done with care due to the decoupled perception-action responses used in these studies, and the coupled perception-action responses in our study, which could possibly lead to divergent performance outcomes (Farrow et al., 2005; Ranganathan and Carlton, 2007; Huesmann et al., 2021).

Overall, our sport-specific motor approach detected distinctions in decision-making between players of varying performance levels. Hence, future analyses of sensorimotor decision making with take-the-first (Johnson and Raab, 2003) and take-the-best (Gigerenzer and Todd, 2001) heuristic models seem promising.

### Limitations and Future Research

We must emphasize that derivations to real-world behavior needs further methodological adjustments in the experimental design.

It still remains open, if the findings obtained in this study reflects the actual on-field behavior of the players. Comparisons of our lab-based performance to the players’ on-field performances during a team-handball match would have allowed for further correlations with the data in our study. The players’ on-field performances in play could have been captured by using a notational system (expert ratings of players’ actions with scores) applied by van Maarseveen et al. (2018a) in soccer. However, a validated team-handball-specific notational system is not existing so far.

Furthermore, players’ physical appearance (height, weight), physical skills (speed, agility), and technical skills (high or low level skills, position-specific skills) are performance-discriminating contextual features in team-handball (Wagner et al., 2014) but, for methodological reasons, these features were standardized in the present study. For example, the physical appearance of a backcourt player also affects defense behavior of a central block player, meaning that a taller backcourt player prefers to make advantage of his height by using long distance throws from the backcourt, whereat smaller players rather prefer Breakthrough actions in near-range distance toward the 6 m line. Consequently, there are common tactical approaches to defend taller backcourt players by early tackling them to avoid long rage throws. For this reason, we decided for a standardized test protocol that minimized such contextual features.

Also, intra-individual differences within the expert group itself (elite players) can also affect the obtained performance outcomes, as revealed by the temporal occlusion study of expert field hockey goalkeepers (Morris-Binelli et al., 2021).

Further experimental investigations are needed to address the influence of contextual priors (Gredin et al., 2020) on decision making in representative task designs. Specifically, future studies could integrate stable priors either verbally with coach-like instructions (typical in a match preparation) about action preferences of a special opponent in the stimuli (see Helm et al., 2020; Lüders et al., 2020) or the tactical direction (see Levi and Jackson, 2018). Dynamic priors in terms of a visual response-depending match status (Farrow and Reid, 2012) as feedback would reinforce the pressure condition during the match. Eye-trackers could gain insights about the utilization of kinematic information in the video stimuli in the test (Dicks et al., 2010; Brams et al., 2019).

Due to the qualitative (response frequency) and quantitative (decision time) variables used in this study, T-pattern analyses, a software-based mixed methods approach, could give additional enlightenment about the temporal structure of the player’s decision behavior (Magnusson, 2000). T-patterns are dendrograms, showing the order and the temporal distributions of occurrences, as well as recurring series of behavioral occurrences. In other words: “if A is an earlier and B a later component of the same recurring T-pattern, then, after an occurrence of A at *t*, there is an interval that tends to contain at least one occurrence of B more often than would be expected by chance” (Magnusson, 2000). Pic (2018) found more T-patterns in home teams compared to away teams in team-handball, meaning that home teams showed repeating patterns of throws in the left and right areas toward the opponent goalkeeper with greater chances for success. Future analyses with this method could explain strategic details and the temporal distributions in defensive decision-making processes, such as repeating slower but better defense responses on specific attack actions.

To conclude, there is accumulating evidence that perceptual-cognitive skills such as decision making constitute a performance-discriminating component in team-sports. The observed expertise effect in response frequency indicates preferences for forward/tackling responses of elite players. Our results are also indicative of *dynamic inconsistency* mechanisms from simple heuristics literature (Raab, 2003; Raab and Johnson, 2007; Raab and Laborde, 2011). Thus, a non-existent expertise effect in decision time may suggest that the required time for making a decision could play a more important role in decision making and simple heuristics than previously assumed. Taken together, our findings serve recent calls in sport science for an enhanced utilization of multidisciplinary test approaches when assessing complex sportive behavior of athletes. Talent detection and identification processes should henceforth apply sport-specific performance tests which take the perceptual-cognitive capabilities of athletes into account. Considering decision making in performance analyses could provide a more holistic estimate of an athletes’ talent and performance potential, as a product of the athletes’ sensory and biomotor capacities at the same time.

## Data Availability Statement

The raw data supporting the conclusions of this article will be made available by the authors, without undue reservation.

## Ethics Statement

The studies involving human participants were reviewed and approved by the Local Ethics Committee from the Otto von Guericke University Magdeburg. Written informed consent to participate in this study was provided by the participants’ legal guardian/next of kin.

## Author Contributions

MT, KM, and MH: conceptualization. NL, MT, and MH: methodology and validation. MH: software, investigation, resources, data curation, and writing – original draft preparation. NL and MH: formal analysis and visualization. MT, HW, NA, JT-C, and NL: writing – review and editing. MT and HW: supervision. MT and KM: project administration and funding acquisition. All authors have read and agreed to the published version of the manuscript.

## Conflict of Interest

The authors declare that the research was conducted in the absence of any commercial or financial relationships that could be construed as a potential conflict of interest.

## Publisher’s Note

All claims expressed in this article are solely those of the authors and do not necessarily represent those of their affiliated organizations, or those of the publisher, the editors and the reviewers. Any product that may be evaluated in this article, or claim that may be made by its manufacturer, is not guaranteed or endorsed by the publisher.
