# Supplementary material for: Differences in Decision-Making Behavior Between Elite and Amateur Team-Handball Players in a Near-Game Test Situation
Source: Front Psychol. 2022 Apr 5;13:854208. doi: 10.3389/fpsyg.2022.854208 (PMC9038659; doi:10.3389/fpsyg.2022.854208)
Supplement: Supplementary file 1 [file Table_1.DOCX]

Supplementary Material

| **Elite** | | **Amateur** | |
| --- | --- | --- | --- |
| **Breakthrough** | | | |
| **A)** | | **B)** | |
|  | |  | |
| **Jump throw** | | | |
| **B)** | | **F)** | |
|  | |  | |
|  | **Standing throw** | |  |
| **C)** | | **G)** | |
|  | |  | |
|  | **Pass** | |  |
| **D)** | | **H)** | |
|  | |  | |
| 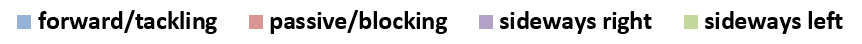 | | | |

**Figure S1**. Frequency distributions of motor responses on all right-handed attacks of elite (left) and amateur players (right) over occlusion time points. Stacked area graphs show occlusion points (x-axis) and response frequency (y-axis), dotted areas indicate significant higher between-group frequency. Colored areas in the graphs illustrate the respective response distribution over time in the elite (**A-D**) and amateur (**E-H**) player group.

**Table S1**. Elite-amateur player comparisons for response frequency at each occlusion time point (t_6_-t_0_) in all right-handed attacks (BT=Breakthrough, JT=Jump throw, ST=Standing throw, PA=Pass). Between-group differences are reported as chi-square statistics (*χ*^2^; *p*) and the corresponding effect size *φ*. Fischer calculation method was not applicable due to non-consistent directional differences in response frequency between the groups. Non available (n.a.) comparisons reflect that the respective motor response did not occur in neither group.

|  |  | **t6** | **t5** | **t4** | **t3** | **t2** | **t1** | **t0** |  |
| --- | --- | --- | --- | --- | --- | --- | --- | --- | --- |
| **BT** |  |  |  |  |  |  |  |  |  |
|  | Passive/  blocking | χ^2^(1) = 0.43,  *p* = .511,  *φ* = .11 | χ^2^(1) = 0.54,  *p* = .463,  *φ* = .12 | χ^2^(1) = 0.00,  *p* = 1.000,  *φ* = .00 | χ^2^(1) =0.28,  *p* = .868,  *φ* = .03 | χ^2^(1) = 3.70,  *p* = .054,  *φ* = .31 | χ^2^(1) = 1.17,  *p* = .279,  *φ* = -.18 | χ^2^(1) = 0.00,  *p* = .955,  *φ* = -.01 |  |
|  | Forward/  tackling | χ^2^(1) = 0.43,  *p* = .511,  *φ* = -.11 | χ^2^(1) = 3.57,  *p* = .059,  *φ* = -.32 | χ^2^(1) = 0.11,  *p* = .735,  *φ* = -.06 | χ^2^(1) = 0.02,  *p* = .966,  *φ* = .01 | χ^2^(1) = 2.18,  *p* = .139,  *φ* = -.24 | χ^2^(1) = 1.28,  *p* = .258,  *φ* = -.19 | χ^2^(1) = 0.12,  *p* = .727,  *φ* = -.06 |  |
|  | Sideways  left | n.a. | n.a. | χ^2^(1) = 1.03,  *p* = .310,  *φ* = -.17 | χ^2^(1) = 5.81,  *p* = .447,  *φ* = -.13 | χ^2^(1) = 2.96,  *p* = .587,  *φ* =-.09 | χ^2^(1) = 2.46,  *p* = .115,  *φ* = .27 | χ^2^(1) = 0.03,  *p* = .955,  *φ* = -.01 |  |
|  | Sideways  right | n.a. | χ^2^(1) = 3.66,  *p* = .056,  *φ* = .32 | χ^2^(1) = 2.12,  *p* = .146,  *φ* = .24 | χ^2^(1) = 1.47,  *p* = .225,  *φ* = .21 | n.a. | χ^2^(1) = 0.87,  *p* = .352,  *φ* = .16 | n.a. |  |
| **JT** |  |  |  |  |  |  |  |  |  |
|  | Passive/  blocking | χ^2^(1) = 0.00,  *p* = .985,  *φ* = -.00 | χ^2^(1) = 0.42,  *p* = .516,  *φ* = -.11 | χ^2^(1) = 0.12,  *p* = .729,  *φ* = -.06 | χ^2^(1) = 0.23,  *p* = .631,  *φ* = .08 | χ^2^(1) = 1.82,  *p* = .177,  *φ* = .23 | χ^2^(1) = 0.25,  *p* = .618,  *φ* = .08 | χ^2^(1) = 2.00,  *p* = .157,  *φ* = .24 |  |
|  | Forward/  tackling | χ^2^(1) = 0.00,  *p* = .985,  *φ* = -.00 | χ^2^(1) = 0.10,  *p* = .758,  *φ* = .05 | χ^2^(1) = 0.00,  *p* = 1.000,  *φ* = .00 | χ^2^(1) = 0.23,  *p* = .631,  *φ* = -.08 | χ^2^(1) = 1.82,  *p* = .177,  *φ* = -.23 | χ^2^(1) = 0.26,  *p* = .618,  *φ* = -.08 | χ^2^(1) = 2.00,  *p* = .157,  *φ* = -.24 |  |
|  | Sideways  left | χ^2^(1) = 0.92,  *p* = .337,  *φ* = -.16 | χ^2^(1) = 1.14,  *p* =.285,  *φ* = .17 | χ^2^(1) = 1.03,  *p* = .310,  *φ* = .17 | n.a. | n.a. | n.a. | n.a. |  |
|  | Sideways  right | χ^2^(1) = 1.15,  *p* = .284,  *φ* = .18 | n.a. | n.a. | n.a. | n.a. | n.a. | n.a. |  |
| **ST** |  |  |  |  |  |  |  |  |  |
|  | Passive/  blocking | χ^2^(1) = 0.40,  *p* = .525,  *φ* = -.10 | χ^2^(1) = 0.03,  *p* = .862,  *φ* = .03 | χ^2^(1) = 0.44,  *p* = .505,  *φ* = -.11 | χ^2^(1) = 0.73,  *p* = .393,  *φ* = -.15 | χ^2^(1) = 2.38,  *p* = .123,  *φ* = -.27 | χ^2^(1) = 0.51,  *p* = .476,  *φ* = .12 | χ^2^(1) = 0.00,  *p* = .985,  *φ* = .00 |  |
|  | Forward/  tackling | χ^2^(1) = 0.10,  *p* = .749,  *φ* = -.05 | χ^2^(1) = 0.02,  *p*= .877,  *φ* = .03 | χ^2^(1) = 0.11,  *p* = .738,  *φ* = .06 | χ^2^(1) = 0.73,  *p* = .392,  *φ* = .15 | χ^2^(1) = 1.55,  *p* = .213,  *φ* = .21 | χ^2^(1) = 0.51,  *p* = .476,  *φ* = -.12 | χ^2^(1) = 0.47,  *p* = .495,  *φ* = -.11 |  |
|  | Sideways  left | χ^2^(1) = 2.06,  *p* = .151,  *φ* = .23 | χ^2^(1) = 0.97,  *p* = .324,  *φ* = -.17 | χ^2^(1) = 1.03,  *p* = .310,  *φ* = .17 | χ^2^(1) = 0.00,  *p* = .965,  *φ* = .01 | χ^2^(1) = 0.81,  *p* = .367,  *φ* = .16 | n.a. | χ^2^(1) = 1.80,  *p* = .180,  *φ* = .22 |  |
|  | Sideways  right | n.a. | n.a. | n.a. | n.a. | n.a. | n.a. | n.a. |  |
| **PA** |  |  |  |  |  |  |  |  |  |
|  | Passive/  blocking | χ^2^(1) = 0.35,  *p* = .114,  *φ* = .09 | χ^2^(1) = 0.47,  *p* = .492,  *φ* = -.12 | χ^2^(1) = 0.35,  *p* = .557,  *φ* = .10 | χ^2^(1) = 0.76,  *p* = .385,  *φ* = .14 | χ^2^(1) = 0.75,  *p* = .386,  *φ* = .14 | χ^2^(1) = 0.74,  *p* = .389,  *φ* = .14 | χ^2^(1) = 0.37,  *p* = .542,  *φ* = .10 |  |
|  | Forward/  tackling | χ^2^(1) = 0.42,  *p* = .516,  *φ* = -.10 | χ^2^(1) = 0.47,  *p* = .492,  *φ* = .12 | χ^2^(1) = 0.35,  *p* = .557,  *φ* = -.10 | χ^2^(1) = 1.51,  *p* = .219,  *φ* = -.20 | χ^2^(1) = 1.64,  *p* = .200,  *φ* = -.21 | χ^2^(1) = 0.60,  *p* = .438,  *φ* = -.13 | χ^2^(1) = 1.06,  *p* = .303,  *φ* = -.17 |  |
|  | Sideways  left | χ^2^(1) = 0.01,  *p* = .916,  *φ* = .02 | n.a. | n.a. | n.a. | χ^2^(1) = 1.14,  *p* = .285,  *φ* = .17 | n.a. | n.a. |  |
|  | Sideways  right | n.a. | n.a. | n.a. | χ^2^(1) = 1.09,  *p* = .298,  *φ* = .17 | n.a. | χ^2^(1) = 0.05,  *p* = .823,  *φ* = -.04 | χ^2^(1) = 0.06,  *p* = .807,  *φ* = .04 |  |
|  |  |  |  |  |  |  |  |  |  |

| **Elite** | | **Amateur** | |
| --- | --- | --- | --- |
| **Breakthrough** | | | |
| **A)** | | **B)** | |
|  | |  | |
| **Jump throw** | | | |
| **B)** | | **F)** | |
|  | |  | |
|  | **Standing throw** | |  |
| **C)** | | **G)** | |
|  | |  | |
|  | **Pass** | |  |
| **D)** | | **H)** | |
|  | |  | |
| 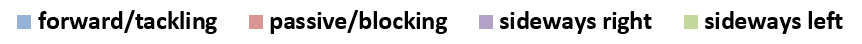 | | | |

**Figure S2.** Frequency distributions of motor responses on all left-handed attacks of elite (left) and amateur players (right) over occlusion time points. Stacked area graphs show occlusion points (*x*-axis) and response frequency (*y*-axis), dotted areas indicate significant higher between-group frequency. Colored areas in the graphs illustrate the respective response distribution over time in the elite (**A-D**) and amateur (**E-H**) player group.

**Table S2.** Elite-amateur player comparisons regarding response frequency at each occlusion time point (t_6_-t_0_) in all left-handed attacks (BT=Breakthrough, JT=Jump throw, ST=Standing throw, PA=Pass). Between-group differences are reported as chi-square statistics (*χ*^2^; *p*) and the corresponding effect size *φ*. Single *p*-values show the results of Fischer method calculations, applicable only when between-group differences in response frequency were directional consistent. Non available (n.a.) comparisons reflect that the respective motor response did not occur in neither group.

|  |  | **t6** | **t5** | **t4** | **t3** | **t2** | **t1** | **t0** | ***p*** |  |
| --- | --- | --- | --- | --- | --- | --- | --- | --- | --- | --- |
| **BT** |  |  |  |  |  |  |  |  |  |  |
|  | Passive/  blocking | χ^2^(1) = 0.44,  *p* = .505,  *φ* = .11 | χ^2^(1) = 0.69,  *p* = .408,  *φ* = .13 | χ^2^(1) = 0.00,  *p* = 1.000,  *φ* = .00 | χ^2^(1) = 1.93,  *p* = .165,  *φ* = .22 | χ^2^(1) = 2.17,  *p* = .140,  *φ* = .24 | χ^2^(1) = 0.32,  *p* = .858,  *φ* = .03 | χ^2^(1) = 1.71,  *p* = .190,  *φ* = .25 | - |  |
|  | Forward/  tackling | χ^2^(1) = 0.44,  *p* = .505,  *φ* = -.11 | χ^2^(1) = 2.31,  *p* = .129,  *φ* = -.24 | χ^2^(1) = 1.91,  *p* = .168,  *φ* = -.22 | **χ^2^(1) = 5.20,**  ***p* = .023,**  *φ* **= -.36** | **χ^2^(1) = 4.67,**  ***p* = .031,**  *φ* **= -.35** | χ^2^(1) = 1.76,  *p* = .189,  *φ* = -.22 | n.a. | **.033** |  |
|  | Sideways  left | n.a. | χ^2^(1) = 2.22,  *p* = .136,  *φ* = .24 | χ^2^(1) = 1.11,  *p* = .292,  *φ* = .17 | n.a. | n.a. | n.a. | n.a. | - |  |
|  | Sideways  right | n.a. | n.a. | χ^2^(1) = 6.25,  *p* = .429,  *φ* = .13 | χ^2^(1) = 0.63,  *p* = .427,  *φ* = .13 | χ^2^(1) = 0.41,  *p* = .839,  *φ =* .03 | χ^2^(1) = 0.18,  *p* = .676,  *φ* = .07 | χ^2^(1) = 1.71,  *p* = .190,  *φ* = -.25 | - |  |
| **JT** |  |  |  |  |  |  |  |  |  |  |
|  | Passive/  blocking | χ^2^(1) = 0.02,  *p* = .894,  *φ* = .02 | χ^2^(1) = 0.61,  *p* = .437,  *φ* = .12 | χ^2^(1) = 1.17,  *p* = .279,  *φ* = .18 | **χ^2^(1) = 4.50,**  ***p* = .034**,  *φ* **= -.35** | χ^2^(1) = 0.67,  *p* = .414,  *φ* = .13 | χ^2^(1) = 0.39,  *p* = .530,  *φ* = -.11 | **χ^2^(1) = 6.80,**  ***p* = .009,**  *φ* **= .43** | **-** |  |
|  | Forward/  tackling | χ^2^(1) = 0.05,  *p* = .821,  *φ* = .04 | χ^2^(1) = 1.18,  *p* = .278,  *φ* = -.17 | **χ^2^(1) = 4.64,**  ***p* = .031,**  *φ* **= -.36** | **χ^2^(1) = 4.50,**  ***p* = .034,**  *φ* **= .35** | χ^2^(1) = 1.48,  *p* = .224,  *φ* = -.19 | χ^2^(1) = 0.20,  *p* = .900,  *φ* = .02 | **χ^2^(1) = 6.80,**  ***p* = .009,**  *φ* **= -.43** | **-** |  |
|  | Sideways  left | n.a. | n.a. | n.a. | n.a. | χ^2^(1) = 0.98, *p* = .323,  *φ* = .15 | n.a. | n.a. | - |  |
|  | Sideways  right | χ^2^(1) = 0.42,  *p* = .517,  *φ* = -.10 | χ^2^(1) = 0.98,  *p* = .323,  *φ* = .15 | **χ^2^(1) = 3.90,**  ***p* = .048,**  *φ* **= .33** | n.a. | n.a. | χ^2^(1) = 1.16,  *p* = .282,  *φ* = .19 | n.a. | - |  |
| **ST** |  |  |  |  |  |  |  |  |  |  |
|  | Passive/  blocking | χ^2^(1) = 0.03,  *p* = .867,  *φ* = .03 | χ^2^(1) = 0.77,  *p* = .380,  *φ* = -.15 | χ^2^(1) = 0.10,  *p* = .758,  *φ* = .05 | χ^2^(1) = 0.65,  *p* = .420,  *φ* = .13 | χ^2^(1) = 2.82,  *p* = .093,  *φ* = .28 | χ^2^(1) = 0.12,  *p* = .914,  *φ* = .02 | χ^2^(1) = 2.20,  *p* = .138,  *φ* = .24 | - |  |
|  | Forward/  tackling | χ^2^(1) = 0.30,  *p* = .585,  *φ* = -.09 | χ^2^(1) = 0.06,  *p* = .803,  *φ* = .04 | χ^2^(1) = 0.39,  *p* = .536,  *φ* = -.10 | χ^2^(1) = 0.65,  *p* = .420,  *φ* = -.13 | χ^2^(1) = 1.27,  *p* = .260,  *φ* = -.19 | χ^2^(1) = 1.64,  *p* = .200,  *φ* = -.21 | χ^2^(1) = 3.72,  *p* = .054,  *φ* = -.32 | - |  |
|  | Sideways  left | n.a. | n.a. | n.a. | n.a. | n.a. | n.a. | n.a. | - |  |
|  | Sideways  right | χ^2^(1) = 0.00,  *p* = .959,  *φ* = .01 | χ^2^(1) = 1.01,  *p* = .316,  *φ* = .17 | χ^2^(1) = 0.92,  *p* = .336,  *φ* = .16 | n.a. | n.a. | n.a. | χ^2^(1) = 1.21,  *p* = .272,  *φ* = .18 | - |  |
| **PA** |  |  |  |  |  |  |  |  |  |  |
|  | Passive/  blocking | χ^2^(1) = 0.03,  *p* = .858,  *φ* = .03 | χ^2^(1) = 3.17,  *p* = .075,  *φ* = .29 | χ^2^(1) = 2.79,  *p* = .095,  *φ* = .28 | χ^2^(1) = 3.75,  *p* = .053,  *φ* = .31 | χ^2^(1) = 2.06,  *p* = .151,  *φ* = .25 | **χ^2^(1) = 4.54,**  ***p* = .033,**  *φ* **= .34** | χ^2^(1) = 0.18,  *p* = .180,  *φ* = .22 | **.013** |  |
|  | Forward/  tackling | χ^2^(1) = 0.29,  *p* = .591,  *φ* = -.09 | χ^2^(1) = 2.17,  *p* = .140,  *φ* = -.24 | χ^2^(1) = 2.79,  *p* = .095,  *φ* = -.28 | χ^2^(1) = 3.75,  *p* = .053,  *φ* = -.31 | χ^2^(1) = 2.06,  *p* = .151,  *φ* = -.25 | **χ^2^(1) = 5.78,**  ***p* =** **.016,**  *φ* **= -.39** | χ^2^(1) = 3.79,  *p* = .052,  *φ* = -.32 | **.001** |  |
|  | Sideways  left | n.a. | χ^2^(1) = 0.96,  *p* = .323,  *φ* = -.16 | n.a. | n.a. | n.a. | χ^2^(1) = 0.01, p = .907,  *φ* = -.02 | χ^2^(1) = 0.40,  *p* = .529,  *φ* = .10 | - |  |
|  | Sideways  right | χ^2^(1) = 1.21,  *p* = .272,  *φ* = .18 | n.a. | n.a. | n.a. | n.a. | n.a. | n.a. | - |  |
|  |  |  |  |  |  |  |  |  |  |  |

**Table S3**. Repeated measures (RM) ANOVA for the decision time in all right- and left-handed attacks. Calculations were made to assess the main effect of the occlusion condition, between-group effects for level, and the group^~^occlusion interactions across occlusion points.

|  | **Offense action** |  | **RM ANOVA** |  | **Right-handed attacks** |  | **Left-handed attacks** |  |
| --- | --- | --- | --- | --- | --- | --- | --- | --- |
|  | Breakthrough |  |  |  |  |  |  |  |
|  |  |  | Main effect |  | ***F*(6, 90) = 4.42, *p* < .001** |  | ***F*(6, 84) = 27.48, *p* < .001** |  |
|  |  |  | Between-group |  | *F*(1, 15) = 0.32, *p* = .581 |  | *F*(1, 14) = 1.39, *p* = .257 |  |
|  |  |  | Group~occlusion |  | *F*(6, 90) = 0.35, *p* = .910 |  | *F*(6, 84) = 1.09, *p* = .374 |  |
|  | Jump throw |  |  |  |  |  |  |  |
|  |  |  | Main effect |  | ***F*(6, 78) = 10.34, *p* < .001** |  | ***F*(6, 96) = 32.51, *p* < .001** |  |
|  |  |  | Between-group |  | *F*(1, 13) = 3.07, *p* = .103 |  | *F*(1, 16) = 0.01, *p* = .945 |  |
|  |  |  | Group~occlusion |  | *F*(6, 78) = 0.68, *p* < .666 |  | *F*(6, 96) = 1.57, *p* < .165 |  |
|  | Standing throw |  |  |  |  |  |  |  |
|  |  |  | Main effect |  | ***F*(6, 96) = 9.52, *p* < .001** |  | ***F*(6, 120) = 18.67, *p* < .001** |  |
|  |  |  | Between-group |  | *F*(1, 16) = 0.00, *p* = .975 |  | *F*(1, 20) = 2.60, *p* = .123 |  |
|  |  |  | Group~occlusion |  | ***F*(6, 96) = 2.33, *p* < .038** |  | *F*(6, 120) = 0.86, *p* < .523 |  |
|  | Pass |  |  |  |  |  |  |  |
|  |  |  | Main effect |  | ***F*(6, 96) = 6.51, *p* < .001** |  | ***F*(6, 114) = 17.29, *p* < .001** |  |
|  |  |  | Between-group |  | *F*(1, 16) = 7.47, *p* = .400 |  | *F*(1, 19) = 0.09, *p* = .771 |  |
|  |  |  | Group~occlusion |  | *F*(6, 96) = 1.46, *p* < .201 |  | *F*(6, 114) = 1.10, *p* < .369 |  |
|  |  |  |  |  |  |  |  |  |
